# Dual nomograms to predict central and lateral cervical lymph node metastasis in papillary thyroid carcinoma with Hashimoto’s thyroiditis: a two-cohort study

**DOI:** 10.3389/fendo.2026.1826515

**Published:** 2026-08-11

**Authors:** KeJie Yu, XianJiang Wu, WeiDong Zhang, Qi Le, Yue Xie, YingChun Wang, Lei Dai

**Affiliations:** Department of Thyroid Surgery, Ningbo No. 2 Hospital, Wenzhou Medical University, Ningbo, Zhejiang, China

**Keywords:** central lymph node metastasis, Hashimoto’s thyroiditis, lateral lymph node metastasis, nomogram, papillary thyroid cancer

## Abstract

**Background:**

In papillary thyroid carcinoma (PTC) patients with suspected Hashimoto’s thyroiditis (HT) indicated by elevated preoperative thyroglobulin antibodies (TgAb) and thyroid peroxidase antibodies (TPOAb), reactive lymphadenopathy can mimic metastatic disease on imaging. This overlap complicates preoperative cervical lymph node assessment and may lead to either unnecessary dissection or missed metastases. We aimed to develop and validate separate nomograms to predict central lymph node metastasis (CLNM) and lateral lymph node metastasis (LLNM) in PTC patients with concurrent HT.

**Methods:**

We retrospectively enrolled 660 PTC patients with preoperatively confirmed HT who underwent thyroid surgery in Ward A of Ningbo No. 2 Hospital (training cohort). Independent predictors of CLNM and LLNM were identified using univariate analyses followed by multivariable logistic regression, and two nomograms were constructed. An independent cohort of 459 patients from Ward B served as an external validation set. Model discrimination, calibration, and clinical utility were assessed using the area under the receiver operating characteristic curve (AUC), calibration plots, and decision curve analysis (DCA), respectively.

**Results:**

For CLNM, age, calcification, capsular status, and maximum tumor diameter (MTD) were independent predictors. For LLNM, tumor margins, capsular status, and MTD were independent predictors. In the training cohort, the CLNM nomogram achieved an AUC of 0.760 and the LLNM nomogram an AUC of 0.864. Both models showed good calibration and favorable net benefit on DCA. External validation confirmed stable performance (AUC 0.726 for CLNM; 0.832 for LLNM).

**Conclusion:**

We developed and externally validated two practical nomograms based on readily available preoperative clinical and ultrasonographic features to estimate CLNM and LLNM risk in PTC patients with concurrent HT. These tools may support preoperative risk stratification and help tailor the extent of neck dissection.

## 1 Introduction

Papillary thyroid carcinoma (PTC) accounts for approximately 80–90% of thyroid cancers and its incidence continues to rise worldwide (1–3). Although the prognosis is generally favorable, cervical lymph node metastasis is common and is associated with locoregional recurrence and poorer disease-free survival (4–6). Accurate preoperative assessment of central lymph node metastasis (CLNM) and lateral lymph node metastasis (LLNM) is therefore essential to guide individualized surgical planning, including decisions about prophylactic central neck dissection and the need for lateral neck evaluation.

Hashimoto’s thyroiditis (HT) is the most prevalent autoimmune thyroid disease and is characterized by lymphocytic infiltration and follicular destruction (7). Clinically, HT is commonly suggested by elevated thyroid peroxidase antibodies (TPOAb) and thyroglobulin antibodies (TgAb) (8–10). TPOAb is considered the most important indicator of HT, being present in approximately 95% of patients, while TgAb testing serves as a valuable complement, providing additional diagnostic support (11). Consequently, preoperative diagnosis of HT primarily relies on the positivity of TPOAb and TgAb, which clinically suggests the presence of HT (12, 13). Numerous studies have demonstrated a close association between HT and the development of PTC, with the coexistence of PTC and HT becoming increasingly common in clinical practice (14). However, the impact of HT on the biological behavior of PTC remains controversial (15). Some studies report a protective association with less aggressive disease (16, 17), whereas others suggest that chronic inflammation may promote tumorigenesis (18, 19). In addition, HT-related reactive lymph node hyperplasia can mimic metastatic nodes on ultrasound, reducing diagnostic specificity (20, 21).

PTC patients with concurrent HT exhibit greater clinical complexity than those with PTC alone, particularly regarding the preoperative assessment of cervical lymph node status (22). In clinical practice, this overlap creates a specific preoperative dilemma: in PTC patients with markedly elevated TgAb and TPOAb levels—suggestive of coexisting HT—the specificity of ultrasound evaluation for cervical lymph nodes is substantially reduced. Reactive lymph nodes in HT often present with cortical thickening, loss of the fatty hilum, or a rounded shape, sonographic features that can overlap with those of metastatic lymph nodes. This overlap leads to increased rates of false-positive or false-negative diagnoses for both CLNM and LLNM (23, 24). As a result, clinicians may either extend surgery unnecessarily, increasing the risk of recurrent laryngeal nerve injury and hypoparathyroidism, or under-treat occult metastases, increasing recurrence risk (25, 26). While many models have been proposed to predict lymph node metastasis in PTC (27–29), few are tailored to patients with concurrent HT, and even fewer provide separate, validated tools for CLNM and LLNM in this population.

Accordingly, we developed and validated two distinct nomograms using routinely available preoperative clinical and ultrasonographic characteristics to estimate the risks of CLNM and LLNM in PTC patients with concurrent HT. Our goal was to provide a practical quantitative tool to improve preoperative risk stratification and support decision-making regarding the extent of lymph node dissection.

## 2 Materials and methods

### 2.1 Patients and data collection

This retrospective study included patients with suspected preoperative HT (TgAb positive and TPOAb positive) who underwent thyroid surgery at the Department of Thyroid Surgery, Ningbo No. 2 Hospital, Wenzhou Medical University, between January 2023 and December 2024, and were postoperatively diagnosed with PTC on histopathology. Preoperative clinical variables (sex, age, body mass index [BMI]) and ultrasonographic features of the primary tumor were collected to evaluate CLNM and LLNM risk. Ultrasound variables included maximum tumor diameter (MTD), multifocality, margins, shape, aspect ratio, calcification, blood flow signal (BFS), and capsular status. The department operates two independent wards. Patients from Ward A formed the training cohort, and patients from Ward B formed the external validation cohort. Preoperative thyroid ultrasound was performed using a high frequency linear probe with the patient supine and neck hyperextended. Images were reviewed independently by two experienced radiologists, with disagreements resolved by consensus with a third senior radiologist.

Variables were defined as follows: multifocality, ≥2 tumor foci within one thyroid lobe; margins, well-defined or ill-defined; shape, regular or irregular; aspect ratio, ≥1 or <1; calcification, presence of hyperechoic foci within the tumor; BFS, intranodular vascularity on color Doppler imaging. Capsular status was categorized as intrathyroidal (IT; confined within the thyroid capsule), close to capsule (CC; tumor-to-capsule distance <1 mm), or capsular invasion (CI; tumor breaching the thyroid capsule).

Surgical extent: central lymph node dissection was performed routinely for all patients, while lateral neck dissection was reserved for those with preoperative imaging findings suggestive of metastasis.

### 2.2 Construction and validation of the prediction model

In the training cohort, univariate analyses were performed to screen candidate predictors for CLNM and LLNM. Variables with statistical significance were entered into multivariable logistic regression to identify independent predictors. Two nomograms were constructed based on the final models, with predictor points proportional to regression coefficients. For each patient, total points were summed to obtain an estimated probability of CLNM or LLNM. External validation was performed in the independent Ward B cohort. Discrimination was assessed by area under the receiver operating characteristic curve (AUC), calibration by calibration plots; and clinical utility by decision curve analysis (DCA).

### 2.3 Statistical analysis

Statistical analyses were performed using SPSS (version 27.0). Group comparisons used χ² tests for categorical variables and appropriate parametric or non-parametric tests for continuous variables. Multivariable logistic regression was used to identify independent predictors. All tests were two-sided, and P<0.05 was considered statistically significant. Forest plots and receiver operating characteristic (ROC) curves were generated in GraphPad Prism (version 10.1.2). Nomograms, waterfall plots, calibration curves, and DCA were produced in R (version 4.4.3).

## 3 Results

### 3.1 Baseline characteristics of the study cohorts

**Figure 1:**
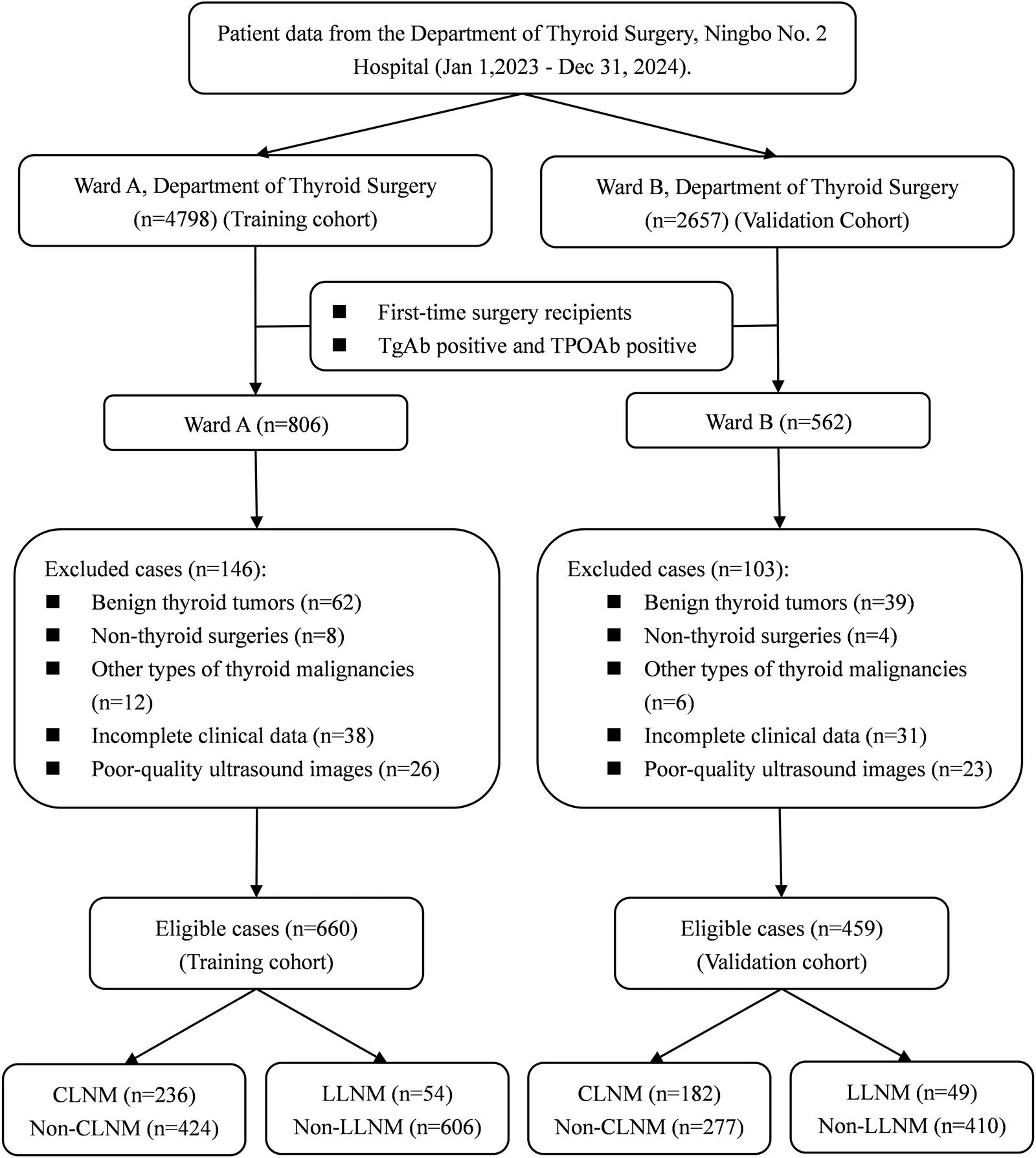
Flowchart of patient selection.

In Ward A, 806 patients with suspected preoperative HT who underwent surgery were screened from 4,798 admissions. We excluded 62 patients with benign thyroid disease, 8 who underwent non-thyroid procedures, 12 with non-PTC malignancies, 38 with incomplete clinical data, and 26 with poor-quality ultrasound images. A total of 660 patients were included in the training cohort (**Figure 1**). CLNM was identified in 236 patients (35.76%) and LLNM in 54 patients (8.18%). Preoperative variables evaluated included sex, age, BMI, MTD, multifocality, margins, shape, aspect ratio, calcification, BFS, and capsular status.

In Ward B, 459 patients were included after screening 2,657 admissions (**Figure 1**). CLNM was present in 182 patients (39.65%) and LLNM in 49 patients (10.68%). Baseline characteristics of both cohorts are summarized in **Table 1**. No statistically significant differences were observed between cohorts for any variable (all P>0.05), indicating good comparability for external validation.

**Table 1:** Baseline comparison of relevant risk factors between Ward A and Ward B.

Characteristic	Training cohort (Ward A) n=660 (%)	Validation cohort (Ward B) n=459 (%)	p-value
Gender			0.185
Male	102 (15.45)	58 (12.64)	
Female	558 (84.55)	401 (87.36)	
Age (years)			0.216
median, IQR	47 (37-57)	46 (36-55)	
BMI (kg/m^2^)			0.064
median, IQR	23.36 (21.10-25.76)	22.86 (20.93-24.97)	
Margins			0.352
Ill-defined	377 (57.12)	275 (59.91)	
Well-defined	283 (42.87)	184 (40.09)	
Shape			0.409
Irregular	483 (73.18)	346 (75.38)	
Regular	177 (26.82)	113 (24.62)	
Aspect ratio			0.056
≥ 1	417 (63.18)	264 (57.52)	
< 1	243 (36.82)	195 (42.48)	
Multifocality			0.641
Yes	106 (16.06)	69 (15.03)	
No	554 (83.94)	390 (84.97)	
Calcification			0.638
Yes	366 (55.45)	248 (54.03)	
No	294 (44.55)	211 (45.96)	
BFS			0.784
Yes	285 (43.18)	195 (42.48)	
No	375 (56.82)	264 (57.52)	
Capsule			0.457
IT	353 (53.48)	234 (50.98)	
CC	263 (39.85)	199 (43.36)	
CI	44 (6.67)	26 (5.66)	
MTD (cm)			0.057
median, IQR	0.66 (0.48-0.93)	0.69 (0.50-0.97)	

BMI, body mass index; BFS, blood flow signals; MTD, maximal tumor diameter; IT, intrathyroidal; CC, capsular contact; CI, capsular invasion.

### 3.2 Selection of predictive factors

#### 3.2.1 Risk factor analysis for CLNM

**Figure 2:**
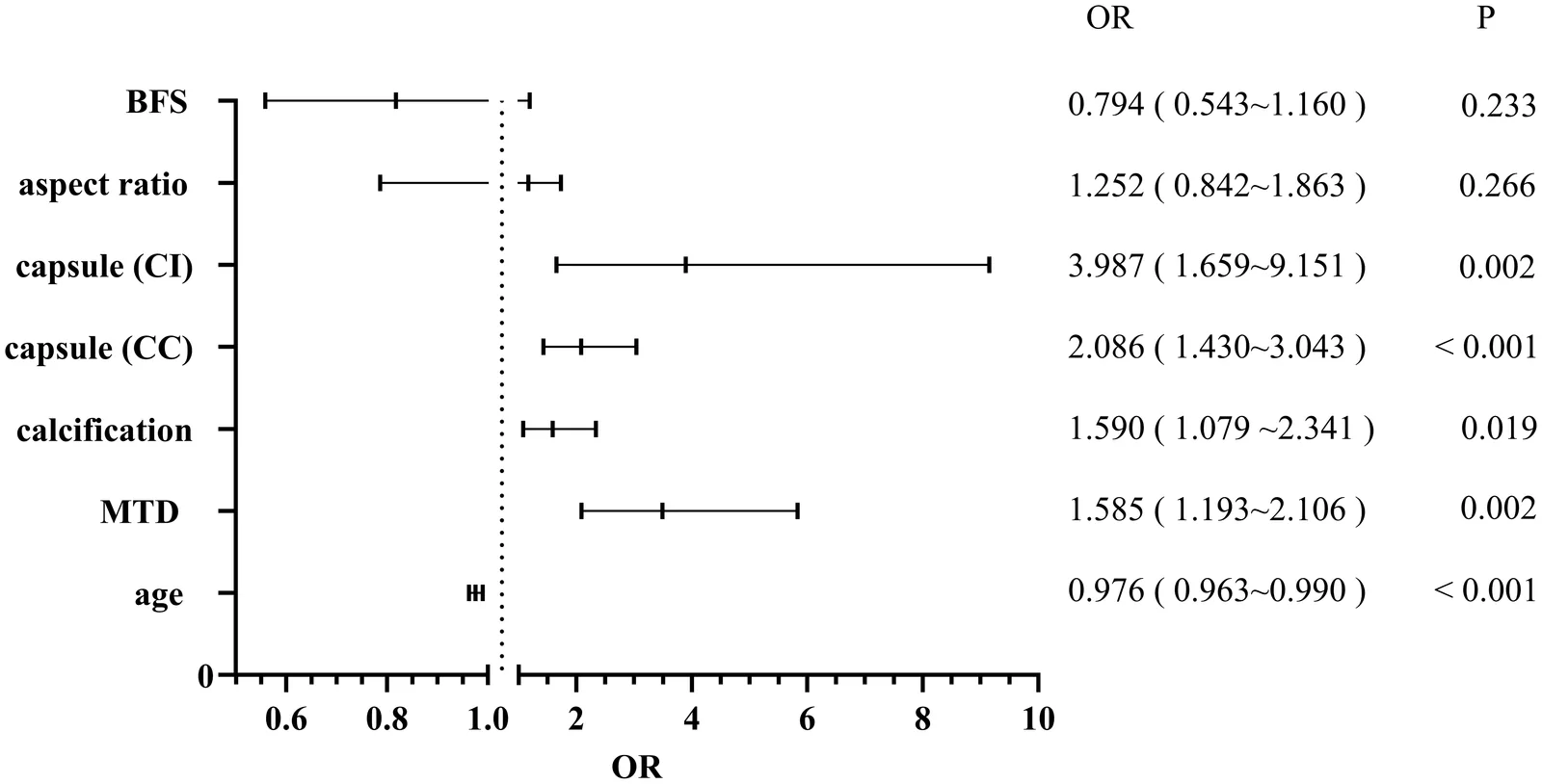
Multivariate logistic regression analysis of risk factors for CLNM from the training cohort.

The 660 patients in Ward A were divided into a CLNM-positive group (n=236) and a CLNM-negative group (n=424). Univariate analyses showed that age (P<0.001), aspect ratio (P = 0.004), calcification (P<0.001), BFS (P = 0.047), capsular status (P<0.001), and MTD (P<0.001) were associated with CLNM (**Table 2**). In multivariable logistic regression, age (OR = 0.976, 95% CI 0.963–0.990; P = 0.001), calcification (OR = 1.590, 95% CI 1.079–2.341; P = 0.019), CC (OR = 2.086, 95% CI 1.430–3.043; P<0.001), CI (OR = 3.897, 95% CI 1.659–9.151; P = 0.002), and MTD (OR = 3.494, 95% CI 2.093–5.833; P<0.001) remained independent predictors (**Figure 2**). Accordingly, age, calcification, capsular status, and MTD were used to build the CLNM model.

**Table 2:** Univariate analysis of risk factors for CLNM in PTC patients with HT from the training cohort.

Characteristic	Number of Patients n=660	CLNM−positive n=236 (%)	CLNM−negative n=424 (%)	p-value
Gender				0.055
Male	102	45 (19.07)	57 (13.44)	
Female	558	191 (80.93)	367 (86.56)	
Age (years)				< 0.001
median, IQR	47 (37-57)	42 (33-55)	50 (39-58)	
BMI (kg/m^2^)				0.227
Median, IQR	23.36 (21.10-25.76)	23.46 (21.45-26.29)	23.33 (21.04-25.39)	
Margins				0.238
Ill-defined	377	142 (60.17)	235 (55.42)	
Well-defined	283	94 (39.83)	189 (44.58)	
Shape				0.546
Irregular	483	176 (74.58)	307 (72.41)	
Regular	177	60 (25.42)	117 (27.59)	
Aspect ratio				0.004
≥ 1	417	132 (55.93)	285 (67.22)	
< 1	243	104 (44.07)	139 (32.78)	
Multifocality				0.365
Yes	106	42 (17.80)	60 (14.15)	
No	554	194 (82.20)	360 (85.85)	
Calcification				< 0.001
Yes	366	168 (71.19)	198 (46.70)	
No	294	68 (28.81)	226 (53.30)	
BFS				0.047
Yes	285	114 (48.31)	171 (40.33)	
No	375	122 (51.69)	253 (59.67)	
Capsule				< 0.001
IT	353	80 (33.90)	273 (64.39)	
CC	263	122 (51.69)	141 (33.25)	
CI	44	34 (14.41)	10 (2.36)	
MTD (cm)				< 0.001
median, IQR	0.66 (0.48-0.93)	0.88 (0.60-1.30)	0.58 (0.46-0.80)	

BMI, body mass index; BFS, blood flow signals; MTD, maximal tumor diameter; IT, intrathyroidal; CC, capsular contact; CI, capsular invasion; CLNM, central lymph node metastasis.

#### 3.2.2 Risk factor analysis for LLNM

**Figure 3:**
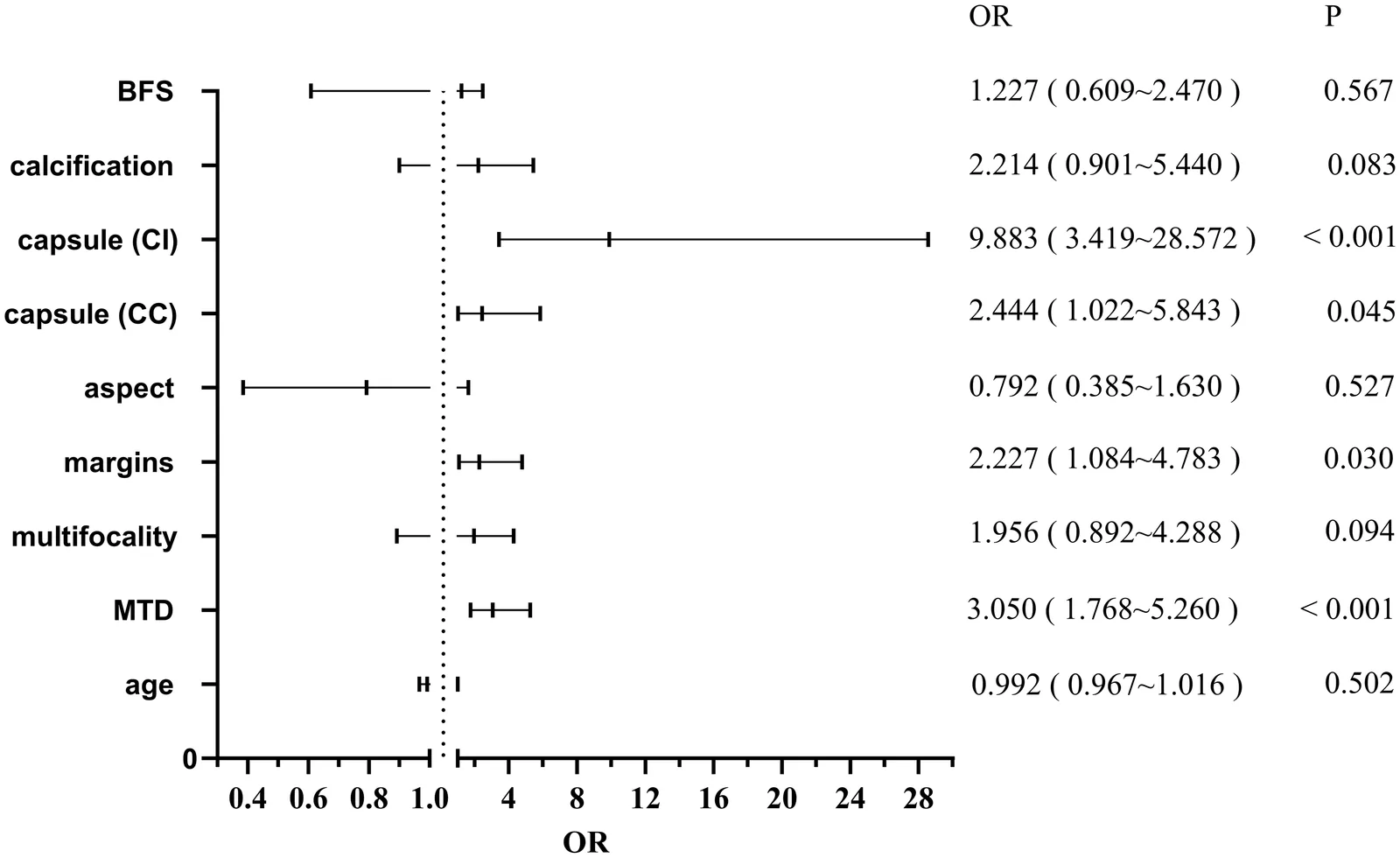
Multivariate logistic regression analysis of risk factors for LLNM from the training cohort.

The cohort was also divided into an LLNM-positive group (n=54) and an LLNM-negative group (n=606). Univariate analyses identified age (P = 0.032), margins (P<0.001), aspect ratio (P<0.001), multifocality (P = 0.039), calcification (P<0.001), BFS (P<0.001), capsular status (P<0.001), and MTD (P<0.001) as potential predictors (**Table 3**). Multivariable logistic regression showed that ill-defined margins (OR = 2.277, 95% CI 1.084–4.783; P = 0.030), CC (OR = 2.444, 95% CI 1.022–5.843; P = 0.045), CI (OR = 9.883, 95% CI 3.419–28.572; P<0.001), and MTD (OR = 3.050, 95% CI 1.768–5.260; P<0.001) were independent predictors (**Figure 3**). Margins, capsular status, and MTD were therefore included in the LLNM model.

**Table 3:** Univariate analysis of risk factors for LLNM in PTC patients with HT from the training cohort.

Characteristic	Number of Patients n=660	LLNM−positive n=54 (%)	LLNM−negative n=606 (%)	p-value
Gender				0.151
Male	102	12 (22.22)	90 (14.85)	
Female	558	42 (77.78)	516 (85.15)	
Age (years)				0.032
median, IQR	47 (37-57)	41 (33-55)	47 (37-57)	
BMI (kg/m^2^)				0.124
median, IQR	23.36 (21.10-25.76)	23.95 (22.03-26.39)	23.32 (21.09-25.65)	
Margins				< 0.001
Ill-defined	377	39 (72.22)	338 (55.78)	
Well-defined	283	15 (27.78)	268 (44.22)	
Shape				0.877
Irregular	483	40 (74.07)	443 (73.10)	
Regular	177	14 (25.93)	163 (26.90)	
Aspect ratio				< 0.001
≥ 1	417	22 (40.74)	395 (65.18)	
< 1	243	32 (59.26)	211 (34.82)	
Multifocality				0.039
Yes	106	14 (25.93)	92 (15.18)	
No	554	40 (74.07)	514 (84.82)	
Calcification				< 0.001
Yes	366	47 (87.04)	319 (52.64)	
No	294	7 (12.96)	287 (47.36)	
BFS				< 0.001
Yes	285	35 (64.81)	250 (41.25)	
No	375	19 (35.19)	356 (58.75)	
Capsule				< 0.001
IT	353	8 (14.81)	345 (56.93)	
CC	263	25 (46.30)	238 (39.27)	
CI	44	21 (38.89)	23 (3.80)	
MTD (cm)				< 0.001
median, IQR	0.66 (0.48-0.93)	1.21 (1.01-2.00)	0.61 (0.47-0.88)	

BMI, body mass index; BFS, blood flow signals; MTD, maximal tumor diameter; IT, intrathyroidal; CC, capsular contact; CI, capsular invasion; LLNM, lateral lymph node metastasis.

### 3.3 Development of the nomogram models

**Figure 4:**
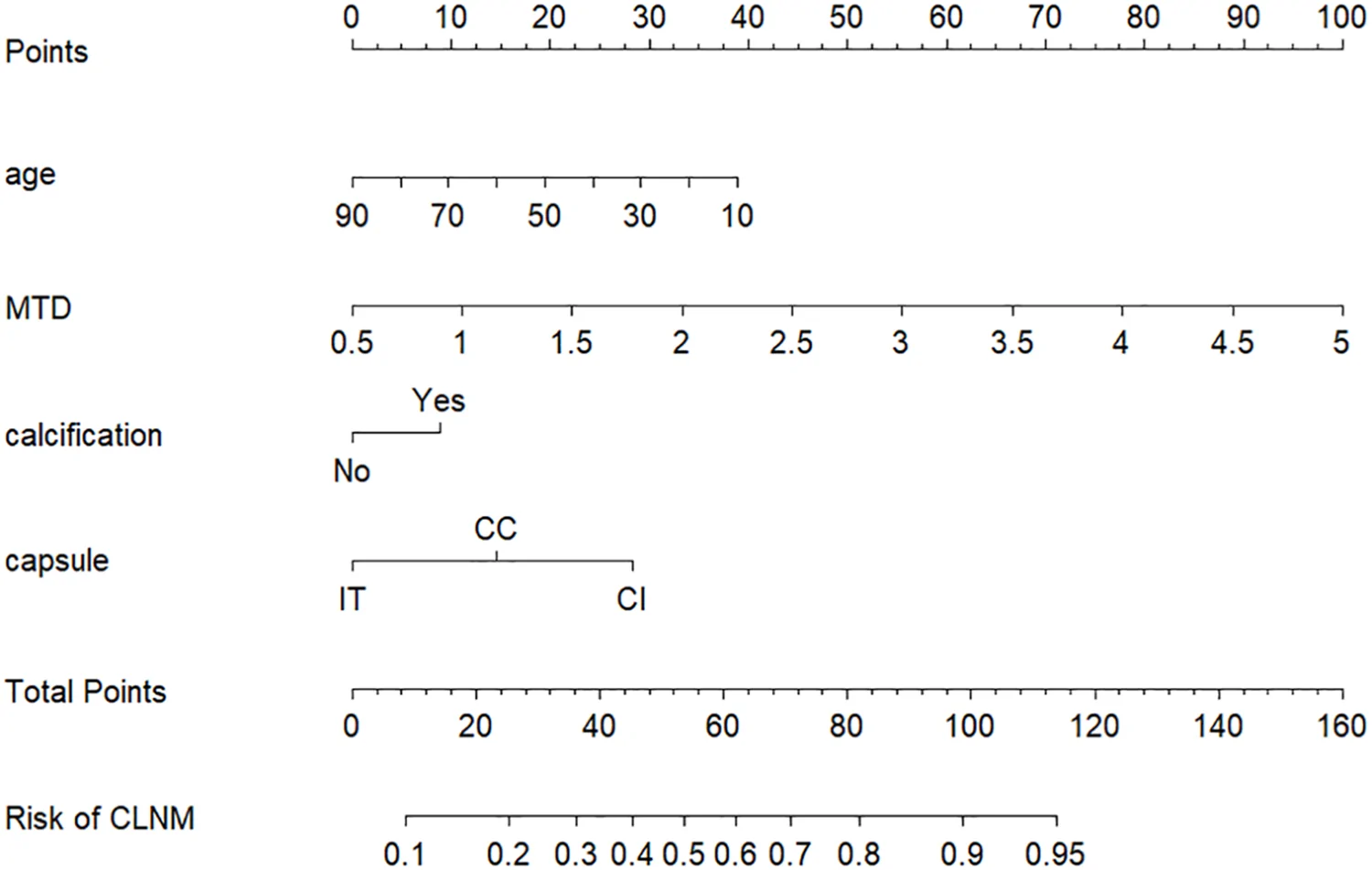
Nomogram for predicting the risk of CLNM.

**Figure 5:**
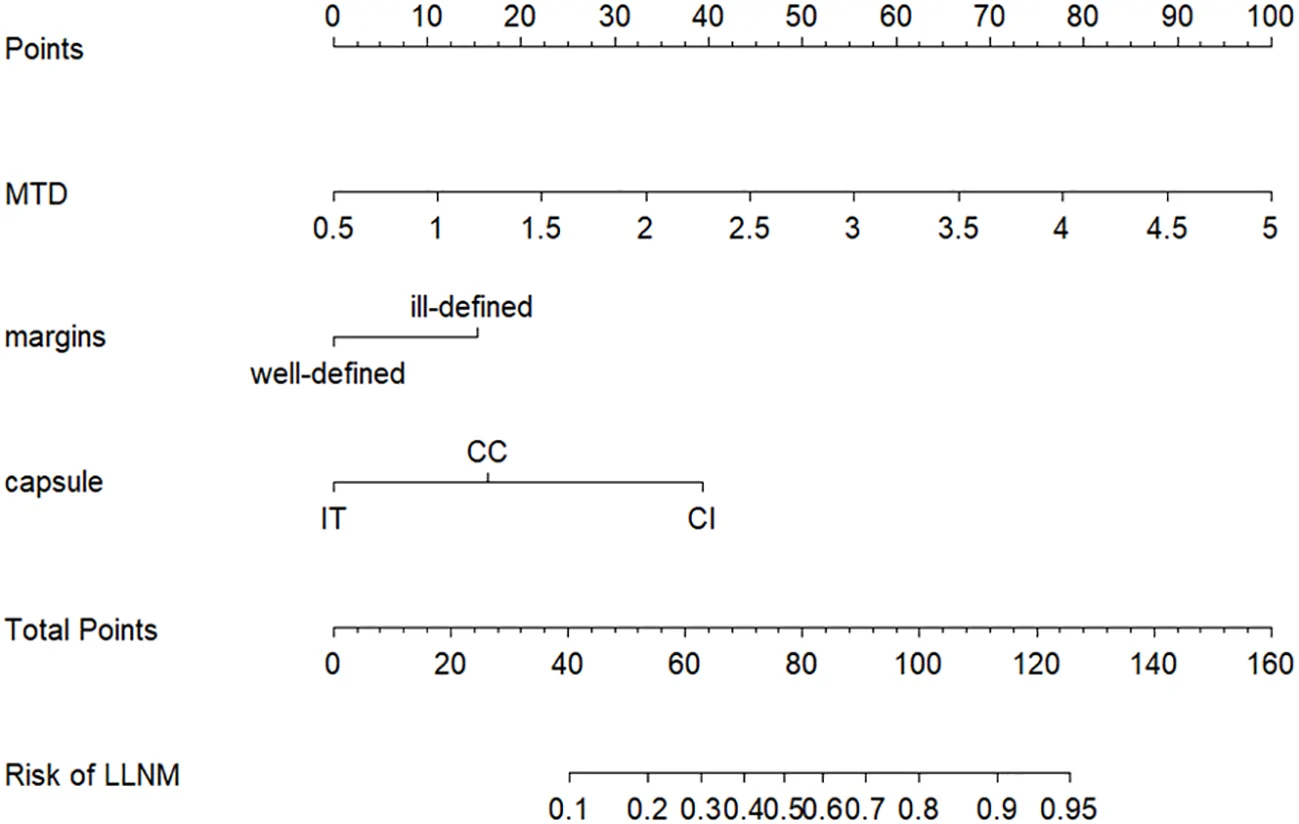
Nomogram for predicting the risk of LLNM.

Two nomograms were constructed based on the independent predictors identified above to estimate individualized probabilities of CLNM and LLNM (**Figures 4**, **5**). In the CLNM prediction nomogram, age ranging from 10 to 90 years corresponded to points decreasing from 38.91 to 0; MTD increasing from 0.5 cm to 5 cm corresponded to points escalating from 0 to 100; the presence of calcification contributed 8.94 points; CC added 14.60 points; and CI added 28.35 points. The Hosmer-Lemeshow test indicated a good model fit (P = 0.137). In the LLNM prediction nomogram, MTD increasing from 0.5 cm to 5 cm corresponded to points escalating from 0 to 100; ill-defined margins contributed 15.30 points; CC added 16.50 points; and CI added 39.41 points. The Hosmer-Lemeshow test also demonstrated a good fit for this model (P = 0.439).

### 3.4 Validation of the nomogram models

**Figure 6:**
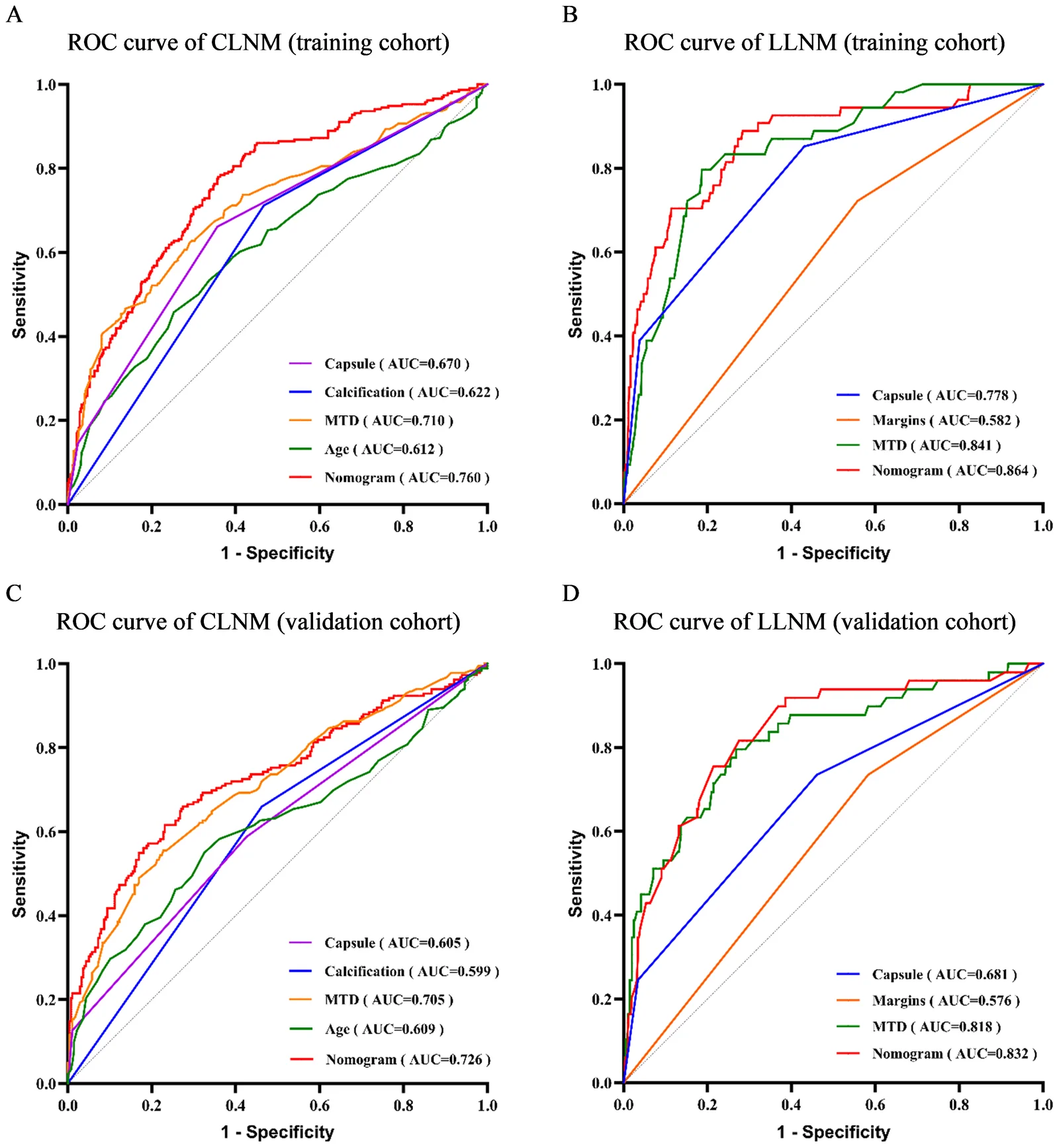
ROC curves of the CLNM and LLNM nomograms in the training and validation cohorts. **(A)** CLNM in the training cohort; **(B)** LLNM in the training cohort; **(C)** CLNM in the validation cohort; **(D)** LLNM in the validation cohort.

**Figure 7:**
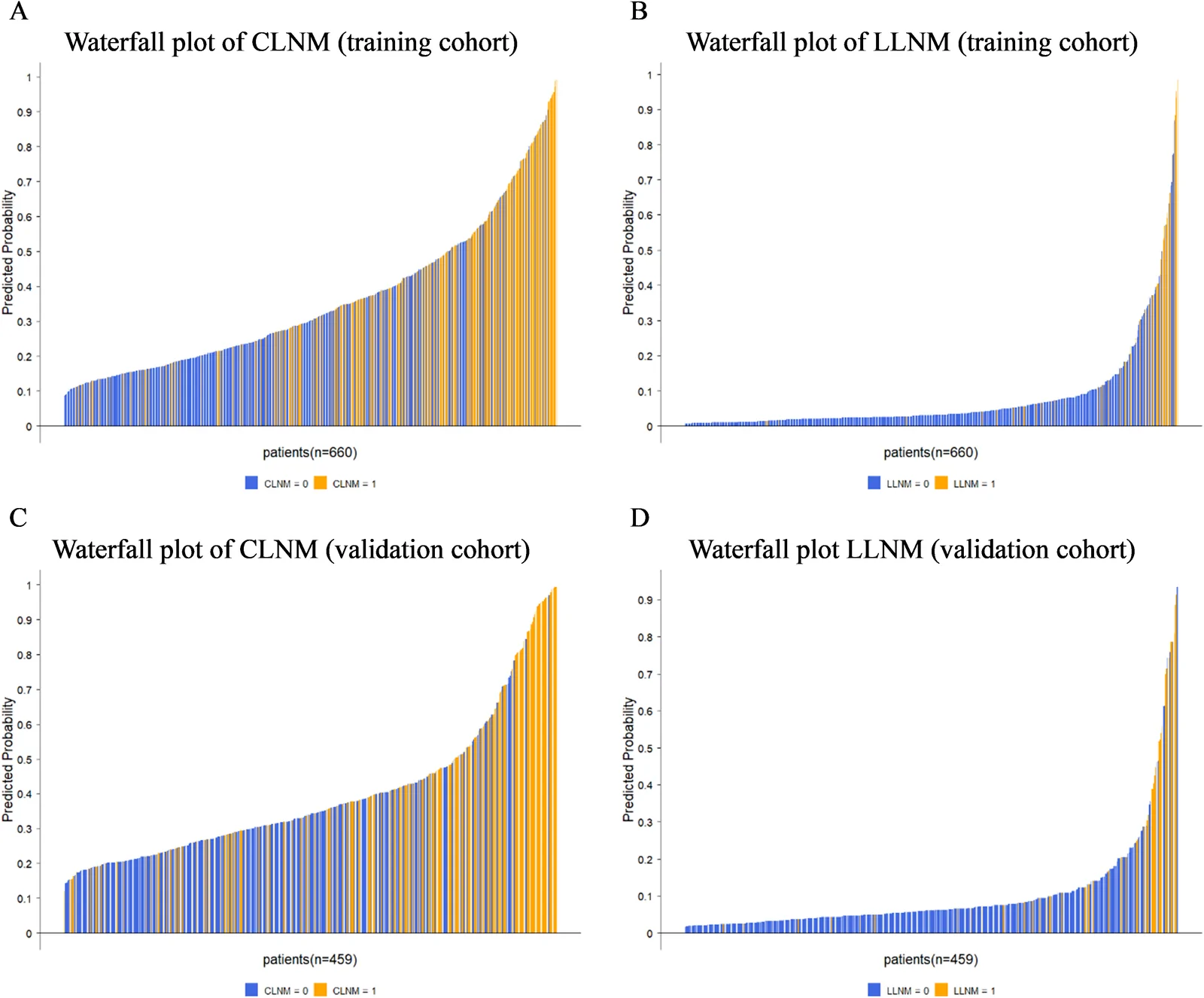
Waterfall plots: distribution of predicted probabilities for **(A)** CLNM in the training cohort; **(B)** LLNM in the training cohort; **(C)** CLNM in the validation cohort; **(D)** LLNM in the validation cohort.

Discrimination was evaluated using ROC curves. In the training cohort, the CLNM nomogram achieved an AUC of 0.760 (95% CI 0.722–0.799) and the LLNM nomogram an AUC of 0.864 (95% CI 0.809–0.918) (**Figures 6A, B**). These results indicate that both nomograms possess good discriminative ability for predicting the presence of CLNM and LLNM, respectively. In the external validation cohort, the CLNM and LLNM nomograms maintained satisfactory discrimination (AUC 0.726, 95% CI 0.677–0.775; and AUC 0.832, 95% CI 0.759–0.886, respectively) (**Figures 6C, D**). Waterfall plots (**Figures 7A–D**) further showed increasing observed CLNM and LLNM rates with increasing predicted probabilities.

**Figure 8:**
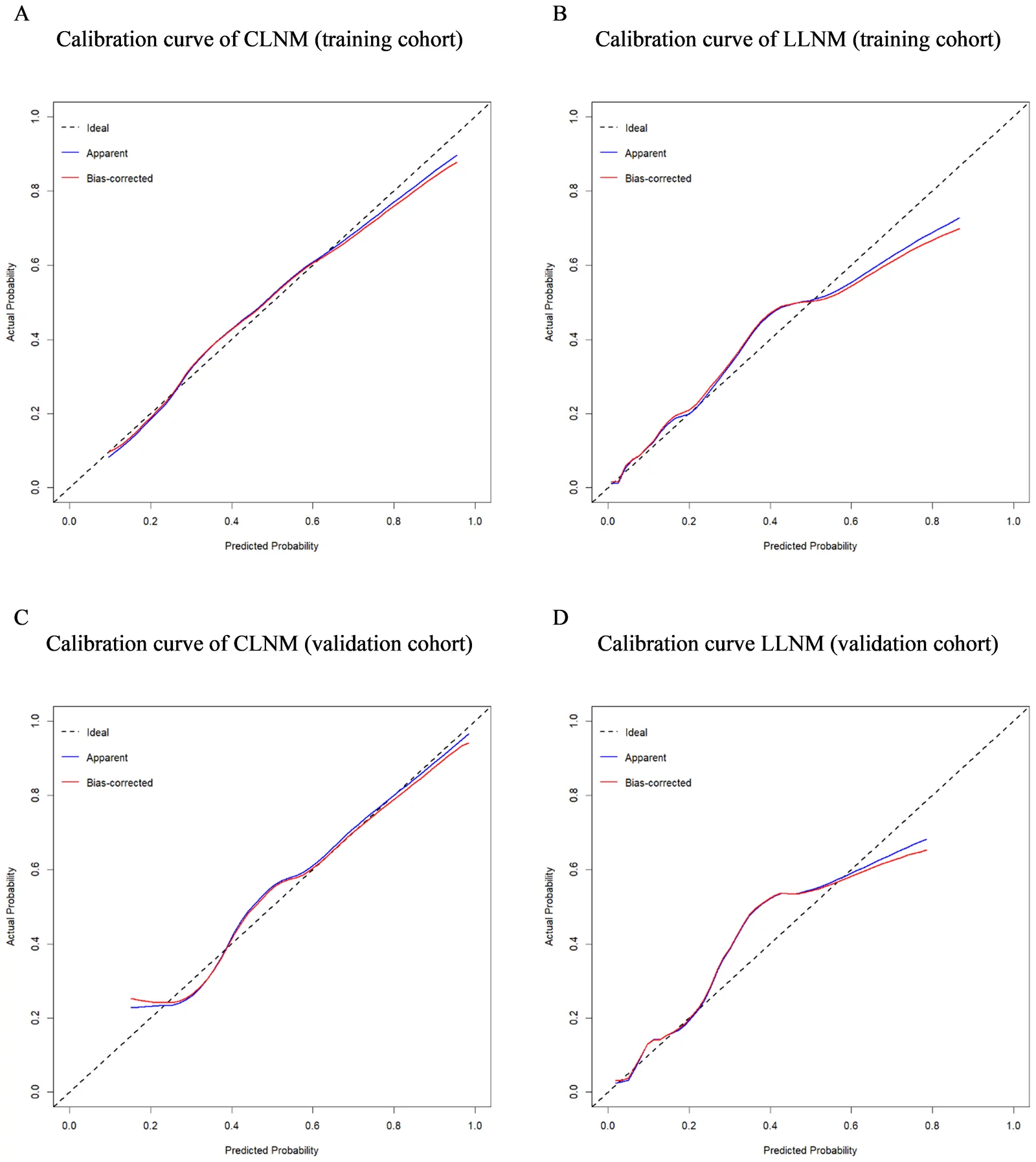
Calibration curves of the nomograms. **(A)** CLNM in the training cohort; **(B)** LLNM in the training cohort; **(C)** CLNM in the validation cohort; **(D)** LLNM in the validation cohort.

**Figure 9:**
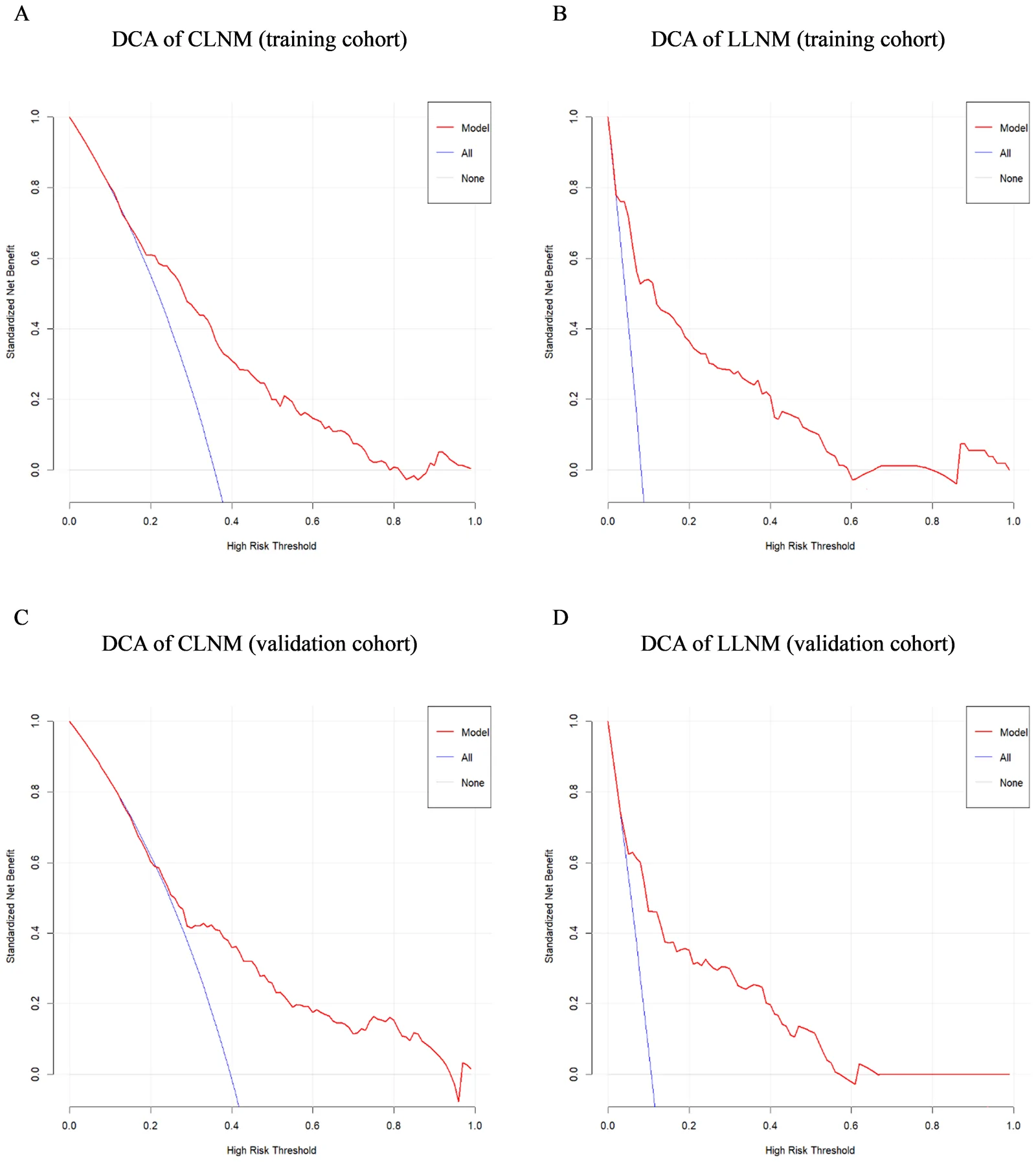
DCA of the nomograms. **(A)** CLNM in the training cohort; **(B)** LLNM in the training cohort; **(C)** CLNM in the validation cohort; **(D)** LLNM in the validation cohort.

To further evaluate the predictive performance of the models, calibration curves were constructed for both the CLNM and LLNM nomograms (**Figures 8A–D**). In both the training and validation cohorts, the apparent and bias-corrected lines closely approximated the ideal diagonal line, indicating excellent calibration. DCA indicated that both nomograms provided net clinical benefit across a wide range of threshold probabilities compared with “treat-all” or “treat-none” strategies (**Figures 9A–D**), supporting their potential clinical utility.

## 4 Discussion

We developed and externally validated two distinct nomograms to predict CLNM and LLNM in PTC patients with concurrent preoperative HT. Because HT can cause reactive lymphadenopathy that mimics metastatic nodes, our models focus on routinely available characteristics of the primary tumor rather than lymph node sonographic features. Age, calcification, capsular status, and MTD independently predicted CLNM, whereas margins, capsular status, and MTD predicted LLNM. Both nomograms demonstrated good discrimination, calibration, and clinical net benefit in the training and validation cohorts, suggesting value for preoperative risk assessment.

### 4.1 Similarities and differences in risk factors for CLNM and LLNM in the context of HT

By developing CLNM and LLNM models within the same PTC population with HT, we identified both shared and distinct predictors for metastasis in different neck compartments. This distinction is clinically relevant because central and lateral metastases differ in lymphatic drainage patterns, surgical morbidity, and typical indications for dissection.

MTD was an independent predictor in both models, consistent with prior reports (30–32). Larger tumors generally reflect greater invasive potential and a longer growth period, increasing the likelihood of lymphatic spread (19, 33, 34). The higher odds ratio for MTD in the LLNM model suggests that increasing tumor size may be particularly informative for anticipating progression to lateral compartment metastasis, which often occurs later in the metastatic cascade. This observation may be explained by the fact that lateral neck metastasis often requires a more extensive tumor burden and a longer time to establish sufficient lymphatic drainage to distant nodal basins, whereas central compartment involvement can occur even with relatively small primary tumors due to the proximity of the central lymph nodes to the thyroid gland.

Capsular status demonstrated predictive value for both types of metastasis, with the probability of metastasis increasing progressively from IT to CC to CI. This finding aligns with the results reported by Lou et al. and Zhao et al. (34, 35), which confirmed that tumor breach of the thyroid capsule directly invades the surrounding lymphatic network, thereby elevating the risk of LNM. However, our study introduced a more nuanced classification by including CC as an intermediate category of capsular status. This refinement also suggests that even when a tumor approaches but has not yet invaded the capsule, the potential for tumor cell dissemination may already be heightened. This incremental risk pattern underscores the clinical importance of precise ultrasonographic assessment of tumor-capsule relationships, as even close proximity may indicate a higher likelihood of early lymphatic spread. Furthermore, capsular status exhibited a more significant predictive value for LLNM than for CLNM. This disparity may be explained by the fact that central compartment lymph nodes, as the first-echelon draining nodes, can harbor micrometastases even in the absence of definitive capsular invasion, whereas lateral compartment involvement requires more extensive local tumor progression and is therefore more dependent on established capsular breach.

Calcification was exclusively incorporated into the CLNM prediction model, consistent with previous studies (36, 37). Calcification, particularly microcalcification, represents a characteristic pathological feature of PTC and is often positively correlated with aggressive tumor behavior (38, 39). The presence of microcalcifications is thought to reflect psammoma bodies, which are concentric calcified structures commonly found in PTC and associated with tumor necrosis, rapid growth, and higher metastatic potential. However, its lack of independent association with LLNM in our cohort may indicate that calcification primarily reflects intrinsic tumor biology, whereas LLNM may depend more strongly on invasive growth characteristics and lymphatic drainage pathways, which are less directly captured by calcification status alone.

Age was an independent predictor for CLNM, with younger patients showing higher central metastasis risk, consistent with large cohort analyses (40). This finding may initially appear counterintuitive, given that HT and PTC are both more prevalent in middle-aged women. However, it is important to distinguish between susceptibility to disease development and the biological behavior of established tumors. Younger patients generally exhibit higher tumor cell proliferative activity and may have more robust immune surveillance, which paradoxically may limit distant hematogenous dissemination while promoting regional lymphatic spread. Additionally, the central compartment serves as the first-echelon drainage basin, making it more susceptible to early micrometastasis in younger patients with more active tumor biology. In contrast, LLNM may be more dependent on local invasive growth and stepwise progression along lymphatic channels than on age alone. This age-related pattern has been observed in several large-scale PTC cohorts and may reflect fundamental differences in tumor biology and host immune response between younger and older patients.

Margins were found to be a predictor exclusively for LLNM. Ill-defined margins are a classic malignant ultrasonographic feature of PTC, reflecting an infiltrative tumor growth pattern. The relationship between margins and lymph node metastasis remains a subject of debate, with current evidence being inconclusive. While some studies have reported that ill-defined margins increase the risk of lymph node metastasis (37, 41, 42), others have excluded margins as a significant risk factor (43, 44). Our study identified a significant association between irregular margins and LLNM, suggesting that tumors with infiltrative growth characteristics are more prone to distant spread along lymphatic vessels. This finding may be explained by the fact that irregular margins indicate more aggressive local invasion, which facilitates tumor cell entry into the perithyroidal lymphatic network and subsequent progression to lateral compartments. The lack of association with CLNM may be due to the central lymph nodes being the first draining station, where even tumors with relatively well-defined margins can still shed cells into the lymphatic system.

### 4.2 Clinical significance and practical value of the models

The two nomograms developed in this study provide clinicians with quantitative tools for individualized preoperative assessment of CLNM and LLNM risk in PTC patients with concurrent HT, based on routinely available clinical and ultrasonographic characteristics. The CLNM nomogram can assist in guiding the necessity of prophylactic central lymph node dissection, thereby helping to avoid unnecessary dissections that carry risks of hypoparathyroidism and recurrent laryngeal nerve injury. The high AUC value of the LLNM nomogram indicates its robust predictive performance. For patients with a high predicted probability of LLNM, intraoperative biopsy of lateral cervical lymph nodes may be warranted; if metastasis is confirmed, lateral neck dissection should be performed. Conversely, for low-risk patients, lateral neck lymph node biopsy may be avoided, reducing surgical trauma. It is important to note that regardless of the model’s prediction score, any lymph node exhibiting highly suspicious sonographic features of metastasis—such as calcification or cystic change—should undergo biopsy or dissection.

### 4.3 Comparison with existing prediction models

Our models have several strengths compared with prior prediction tools. First, HT status was defined using preoperative serology (TgAb and TPOAb), enhancing real-world preoperative applicability rather than relying on postoperative histopathology. Second, we constructed separate models for CLNM and LLNM, reflecting the distinct clinical decisions required for the central and lateral neck. Third, external validation using an independent ward cohort supports the reproducibility and generalizability of the models within the same institution.

### 4.4 Limitations

Several limitations warrant consideration. First, this was a single-center retrospective study, and selection bias cannot be excluded; multicenter prospective validation is needed. Second, although Ward A and Ward B are independent clinical units, both cohorts originated from the same institution. Therefore, the validation performed using Ward B should be regarded as internal rather than true external validation, which may limit the generalizability of our findings. Third, we used univariate analysis for initial variable screening before multivariable logistic regression, which may have limitations in fully capturing important predictors. More robust methods, such as LASSO regression, will be considered in future studies. Fourth, lateral neck dissection was performed only when preoperative ultrasound or CT suggested metastasis, so occult LLNM may have been underestimated.

## 5 Conclusion

We developed and externally validated two nomograms based on routinely available preoperative clinical and ultrasonographic features to predict CLNM and LLNM risk in PTC patients with concurrent HT. Both models showed good discrimination, calibration, and clinical net benefit. These tools may facilitate preoperative risk stratification and support individualized decisions regarding the extent of cervical lymph node assessment and dissection.

## Data Availability

The raw data supporting the conclusions of this article will be made available by the authors, without undue reservation.
